# Add-on quetiapine in the treatment of major depressive disorder in elderly patients with cerebrovascular damage

**DOI:** 10.1186/1745-0179-3-28

**Published:** 2007-11-26

**Authors:** Mauro Giovanni Carta, Fausta Zairo, Gisa Mellino, Maria Carolina Hardoy

**Affiliations:** 1 Department of Public Health, University of Cagliari, Cagliari, Italy; 2 University Center for Research and Clinical Practice in Mental Health, University of Cagliari, Cagliari, Italy; 3 ASL 7 Iglesias (Iglesias), Italy

## Abstract

**Background:**

Depressive episodes in elderly patients with cerebrovascular damage are characterized by poor responses to standard antidepressants. Recent reports have suggested that the atypical antipsychotic, quetiapine may have antidepressant properties and, in mice, may prevents memory impairment and hippocampus neurodegeneration induced by global cerebral ischemia.

**Objective:**

To evaluate the efficacy of combination therapy with quetiapine in depressed elderly patients with cerebrovascular damage.

**Methods:**

An open-label, 6-month follow-up study of patients with major depressive disorder (DSM-IV) and cerebral abnormalities (assessed by MRI) without severe cognitive impairment. Patients who had not responded to standard antidepressants (months of treatment 6.5 ± 7.2) additionally received quetiapine (300 ± 111 mg/d). Patients were evaluated at baseline (t0) and Months 1, 3, and 6 (t1, t3, t6) using the Clinical Global Impressions Scale for Severity (CGI-S) and the Hamilton Depression Rating Scale (HAM-D).

**Results:**

Nine patients were included in the study, with a mean age of 72.8 ± 9.4 years. CGI-S scores decreased from baseline to Month 6: 5.8 ± 0.7 (t0), 5.4 ± 0.7 (t1), 5.0 ± 0.8 (t3), and 4.5 ± 1.0 (t6), with a significant improvement at 6 months compared with baseline (*P *= 0.006). A significant improvement over the 6-month period was also observed with HAM-D scores (t0 = 27.2 ± 4.0, t6 = 14.8 ± 3.8, *P *< 0.001).

**Conclusion:**

In this study, quetiapine was efficacious as combination therapy in depressed elderly patients with cerebrovascular damage. The promising results from this study warrant confirmation in large, randomized, double-blind, placebo-controlled studies.

## Introduction

**Figure 1:**
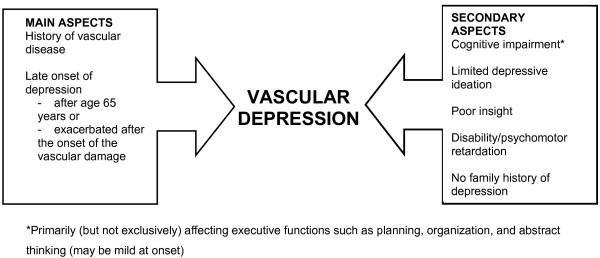
Clinical characteristics of vascular depression.

A serious and common risk to the elderly is depression, which, if untreated, is associated with a high rate of relapse, an increased likelihood of chronicity, and an elevated rate of mortality [1]. Affective disorders (such as depression) and vascular disease (including heart disease) are frequently comorbid conditions that share certain etiopathogenetic and prognostic factors. If untreated, depressive episodes may worsen the course of vascular disease (particularly cerebrovascular diseases) and compromise both quality of life and lifespan expectation. The close correlation between these comorbidities recently led to the identification of so-called "vascular depression" (Figure 1) [2].

Depressive episodes in elderly patients with cerebrovascular damage are characterized by low response rates to antidepressants, and it has therefore become increasingly important to investigate new treatments [3]. However, few therapeutic choices have been validated by strong clinical evidence.

Quetiapine is an atypical antipsychotic approved for the treatment of schizophrenia and episodes of mania associated with bipolar disorder. A lot of studies have also described quetiapine monotherapy to be effective and well tolerated in unipolar [4] and bipolar depression [5]. Recently was reported that quetiapine prevents memory impairment and hippocampus neurodegeneration induced by global cerebral ischemia in mice [6] and pre-administration of quetiapine significantly alleviated the depressive and anxiolytic-like behavioural changes induced by global general ischemia in mice [7]. Authors say that these results suggest a wider perspective for the clinical use of quetiapine.

Nevertheless the US Food & Drug Administration (FDA) advises there may be an increased risk of mortality (mainly due to cardiovascular or infectious causes) in elderly patients with dementia-related psychosis treated with atypical antipsychotics.

## Objective

To evaluate the effectiveness of quetiapine as add-on therapy in elderly patients with late-onset depression and cerebrovascular damage.

## Methods

### Study design

An open-label study of depressed elderly patients resistant to ongoing treatments with cerebrovascular damage who were observed for up to 6 months during add-on treatment with quetiapine.

### Study population

Elderly patients (>65 years of age), with a diagnosis of Major Depressive Disorder (DSM-IV) [8] and cerebrovascular damage documented by magnetic resonance imaging (MRI), no cognitive impairment (Mini Mental State Examination [MMSE] score <25) [9].

Absence of psychotic symptoms or evident psychomotor agitation.

No response to commonly prescribed antidepressants (Hamilton Depression Rating Scale [HAM-D] score >18) [10] following at least 3 months of treatment.

Written consent for the study was obtained after giving patients a complete description of the study.

### Study medication

Quetiapine was administered as add-on therapy with commonly prescribed antidepressants (paroxetine, citalopram, sertraline, mirtazapine).

Quetiapine therapy was initiated at a minimum daily dose of 25 mg/d on Day 1 and was titrated up to 200 mg/d on Day 7 according to the schedule shown in Table 1.

**Table 1:** Titration schedule

Day	1	2	3	4	5	6	7
**Quetiapine dose (mg/d)**	25	25	50	75	100	150	200

After Day 7, the dosage was increased by 100 mg every 2 days until the optimal dose, based on individual response and tolerability, was reached.

### Efficacy assessments

Efficacy was evaluated using the Clinical Global Impression-Severity scale (CGI-S) [11] and HAM-D rating scale [10].

Patients were assessed at baseline (t0), and at the 1-month (t1), 3-month (t3), and 6-month (t6) follow-up visits.

### Statistical methods

Multivariate analysis of variance (MANOVA) was used to test for differences in mean CGI-S and HAM-D scores over time.

## Results

### Patient and treatment characteristics

Nine patients (6 females, 3 males) who had not responded to standard antidepressants (mean [± SD] 6.5 ± 7.2 months of treatment) were included in the study.

Patients had a mean age of 72.8 ± 6.4 years, a mean baseline CGI-S score of 5.8 ± 0.7, and a mean baseline HAM-D score of 27.2 ± 4.0.

Antidepressants administered in combination with quetiapine are shown in Table 2. Other relevant medications taken were benzodiazepines (6 patients) and gabapentin (1 patient).

**Table 2:** Antidepressants used in combination with quetiapine*

Antidepressant	Number of Patients
Paroxetine	3
Citalopram	3
Sertraline	1
Mirtazapine	2

*Some patients received more than one of the listed medications

The mean quetiapine dose (SD) during the study was 300 ± 111 mg/day.

### Efficacy

**Figure 2:**
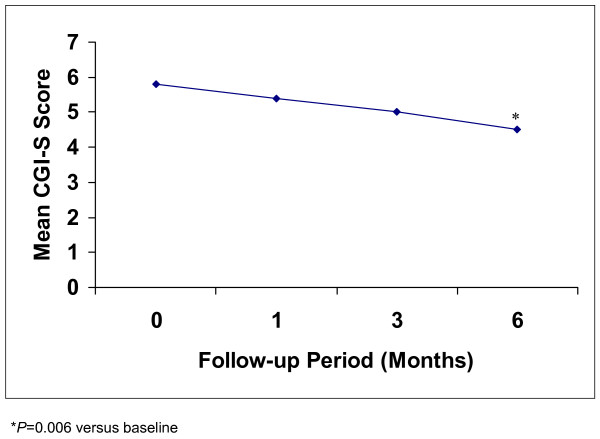
Mean CGI-S scores in elderly patients with depressive episodes and cerebrovascular damage during add-on quetiapine therapy.

During the period of add-on quetiapine treatment, CGI-S scores improved significantly from a mean baseline (t0) score of 5.8 ± 0.7 to 4.5 ± 1.0 at 6 months (t6) (F = 10.21, *P *= 0.006; Table 3, Figure 2).

**Table 3:** Mean CGI-S scores during add-on quetiapine treatment

Evaluation time	Mean score (SD)
Baseline (t0)	5.8 (0.7)
1 month (t1)	5.4 (0.7)
3 months (t3)	5.0 (0.8)
6 months (t6)	4.5 (1.0)

Reductions in mean HAM-D scores from baseline to Month 6 were also statistically significant: t0 = 27.2 ± 4.0, t6 = 14.8 ± 3.8, (F = 34.4, *P *< 0.001).

### Tolerability

No patients discontinued the study.

Side effects reported by patients during the period of add-on quetiapine treatment were sedation (2 patients) and drowsiness (1 patient).

## Discussion and conclusion

Add-on quetiapine therapy significantly improved depressive symptoms and was well tolerated in these elderly patients with comorbid depression and cerebrovascular damage who had previously failed to respond to standard antidepressants.

Although limited by its open-label design and small sample size, this study demonstrates the efficacy and tolerability of quetiapine in this elderly patient population and is consistent with our previous findings [12] and other positive studies of quetiapine in Bipolar Depression [5], Major Depressive Disorder [4] and Generalized Anxiety Disorder [13].

The results of a survey in mice suggest that quetiapine may have defending effects on the impairments induced by cerebral ischemia [6]. Another study shows that quetiapine significantly attenuates bilateral common carotid artery occlusion induced spatial memory impairment and this improvement parallels the alleviative effects of quetiapine on bilateral common carotid artery occlusion induced neurodegeneration in the hilus of hippocampus [7]. Quetiapine may have a neuroprotective and neurogenetic role and this may be related to the therapeutic effects of quetiapine on cognitive deficits in patients with schizophrenia and depression, in which the structure and functions of the hippocampus are implicated [6,7]. The neuroprotective effect of quetiapine was recently confirmed in humans with bipolar disorders [14].

Quetiapine (with clozapine and risperidone) is the drug most commonly used for treat behavioural problems of dementia patients and do not seem to cause severe side effects according to published data, thus may have a possible role in vascular depression [15-17].

These results require confirmation from large, randomized, double-blind, placebo-controlled studies.
